# Systematic evaluation of computational tools to predict the effects of mutations on protein stability in the absence of experimental structures

**DOI:** 10.1093/bib/bbac025

**Published:** 2022-02-21

**Authors:** Qisheng Pan, Thanh Binh Nguyen, David B Ascher, Douglas E V Pires

**Affiliations:** Computational Biology and Clinical Informatics, Baker Heart and Diabetes Institute, Melbourne, Victoria 3004, Australia; School of Chemistry and Molecular Biosciences, University of Queensland, Brisbane City, Queensland 4072, Australia; Systems and Computational Biology, Bio21 Institute, University of Melbourne, 30 Flemington Rd, Parkville, Victoria 3052, Australia; Computational Biology and Clinical Informatics, Baker Heart and Diabetes Institute, Melbourne, Victoria 3004, Australia; School of Chemistry and Molecular Biosciences, University of Queensland, Brisbane City, Queensland 4072, Australia; Systems and Computational Biology, Bio21 Institute, University of Melbourne, 30 Flemington Rd, Parkville, Victoria 3052, Australia; Computational Biology and Clinical Informatics, Baker Heart and Diabetes Institute, Melbourne, Victoria 3004, Australia; School of Chemistry and Molecular Biosciences, University of Queensland, Brisbane City, Queensland 4072, Australia; Systems and Computational Biology, Bio21 Institute, University of Melbourne, 30 Flemington Rd, Parkville, Victoria 3052, Australia; Department of Biochemistry, University of Cambridge, 80 Tennis Ct Rd, Cambridge CB2 1GA, UK; Computational Biology and Clinical Informatics, Baker Heart and Diabetes Institute, Melbourne, Victoria 3004, Australia; School of Chemistry and Molecular Biosciences, University of Queensland, Brisbane City, Queensland 4072, Australia; Systems and Computational Biology, Bio21 Institute, University of Melbourne, 30 Flemington Rd, Parkville, Victoria 3052, Australia; School of Computing and Information Systems, University of Melbourne, Melbourne, Victoria 3053, Australia

**Keywords:** AlphaFold2, mutation effects on protein stability, homology modelling, performance evaluation

## Abstract

Changes in protein sequence can have dramatic effects on how proteins fold, their stability and dynamics. Over the last 20 years, pioneering methods have been developed to try to estimate the effects of missense mutations on protein stability, leveraging growing availability of protein 3D structures. These, however, have been developed and validated using experimentally derived structures and biophysical measurements. A large proportion of protein structures remain to be experimentally elucidated and, while many studies have based their conclusions on predictions made using homology models, there has been no systematic evaluation of the reliability of these tools in the absence of experimental structural data. We have, therefore, systematically investigated the performance and robustness of ten widely used structural methods when presented with homology models built using templates at a range of sequence identity levels (from 15% to 95%) and contrasted performance with sequence-based tools, as a baseline. We found there is indeed performance deterioration on homology models built using templates with sequence identity below 40%, where sequence-based tools might become preferable. This was most marked for mutations in solvent exposed residues and stabilizing mutations. As structure prediction tools improve, the reliability of these predictors is expected to follow, however we strongly suggest that these factors should be taken into consideration when interpreting results from structure-based predictors of mutation effects on protein stability.

## Introduction

Proteins are considered metastable, with their stability intricately linked to their structure and function. Small changes in the protein sequence can lead to large effects on overall protein stability and dynamics, and have been associated with a range of genetic diseases [1–15] and even drug resistance [16–33]. The ability to accurately predict these effects has broad potential applications, not only in interpreting the molecular mechanisms of novel variants [34–36], but also in the industry, where the design of more stable enzymes is of significant importance [37–40].

Experimental approaches to measuring the impact of changes in protein sequence to protein stability, for example thermal melts and urea denaturation, have focussed on measuring the change in Gibbs Free Energy (ΔΔG, expressed in Kcal/mol) of folding by comparing the stability of the purified wild-type and mutant proteins [41]. Although these approaches provide direct experimental insight into protein stability, they are costly and time consuming. This makes it prohibitive to test the effects of every possible mutation and combination of mutations experimentally, and hence, has driven interest in computational approaches to guide more rational mutation analysis and design.

Over the last 20 years, a range of computational approaches have been developed for large-scale studies of the effects of mutations on protein thermodynamics stability. Although they have used a range of different approaches, including statistical [42,43], machine learning [44–54] and energy calculations [55–57], the vast majority have relied upon experimentally solved 3D structures and biophysical measurements in their development [58].

Determination of protein structures, however, is not always straightforward, with a significant number of protein structures yet to be determined experimentally. In the absence of experimental information, homology modelling [59,60] has been widely used to build a 3D model of a protein from its amino acid sequence based on an alignment with a similar protein with known structure, or template. In general, the higher the sequence identity to the template, the more reliable the homology model is likely to be [61]. Although homology models have been widely used to guide interpretation of the effects of mutations using these structure-based tools, it has not been well-established how inaccuracies introduced during the homology modelling affect their reliability and accuracy. In addition to template-based approaches for protein structure prediction, more recently, the development of AlphaFold2 [62] has revolutionized the field with a significant increase in performance, promising to bridge the gap in protein 3D information with high-quality models [63]. Understanding how the use of these protein models affect predictive performance is important to ensure that they are used and interpreted appropriately.

To address this, we have systematically evaluated the effect of homology models on the performance of ten publicly available computational tools for predicting the effects of missense mutations on protein stability.

## Materials and methods

**Figure 1:**
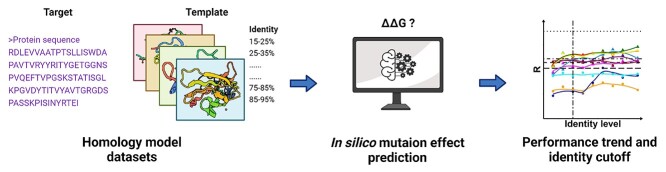
Analysis workflow for assessing the performance of mutation effect predictors using homology models in different sequence identity ranges.

The methodology for the present work can be divided into four main steps (depicted in Figure 1): (i) data acquisition, including collecting experimentally solved protein structures and effects of missense mutation on protein stability; (ii) generating homology models for different identity ranges; (iii) predicting effects of mutations using generated models for a range of available tools; and (iv) comparative analysis of predictive tools.

### Mutation dataset linking effects on stability to experimental protein structures

Over the years, considerable efforts have been dedicated to extracting and manually curating experimentally derived protein thermodynamics information from the literature, including the effects of mutations on stability [64–67] and interactions [68,69], and linking these to high-resolution protein structures. The dataset used in this work was derived from ProTherm [64], and links experimentally measured thermodynamics effects of missense mutations to a diverse set of protein structures. A subset of ProTherm, the S2648 dataset [70], was selected and is composed of 2648 single point missense mutations across 132 unique globular proteins, with a range of mutation effects (Supplementary Figure S1) expressed as the difference in Gibbs Free Energy (ΔΔG) between wild-type (ΔG_WT_) and mutant (ΔG_MT_) as follows:

ΔΔG = ΔG_WT_ − ΔG_MT_

Where positive values denote mutations leading to increased protein stability, while negative values denote destabilizing mutations. Mutations on S2648 are mapped to protein structures solved by either Crystallography with X-ray diffraction or nuclear magnetic resonance (NMR), with 77% of mutations leading to a decrease in protein stability, as observed previously and in other datasets [49,71]. This dataset has been extensively used over the past decade as a benchmark for computational method development aiming to assess mutation effects on stability [44,46,47,70] and, therefore, has been extensively curated and manually inspected. Although this dataset has been used for development purposes before by different methods, the overarching goal of this work is not to assess global performance of the available methods, but rather evaluate how they cope in the absence of experimental structures, when presented with homology models built at different identity thresholds, and how this would impact their relative performance.

### Generating homology models at varied identity levels

In homology modelling, it is well-established that there is a correlation between template identity level and the reliability and quality of models generated [61], despite recent advances in the field pushing the boundaries of both *de novo* [72] and template-free modelling [73]. In order to assess the robustness of currently available predictive methods to input uncertainty and noise, we compared their performance presenting them with the same mutation dataset mapped to homology models built using templates at different sequence identity levels. To achieve this, template candidates for the 132 proteins contained in the S2648 dataset were divided into eight groups, with target-template sequence identities in the following ranges: 15–25%, 25–35%, 35–45%, 45–55%, 55–65%, 65–75%, 75–85% and 85–95%. In addition, performance on the experimental structures was used as upper baseline (e.g. 100% identity dataset). A summary of the developed datasets is shown in Table 1.

**Table 1:** Distributions of the structure- and sequence-based properties of homology model and experimental structure datasets

Identity	# PDBs	# mutations	RSA		Residue depth		Secondary structure types				Secondary structure composition based on CATH			Change of residue volume		
			Buried (%)	Exposed (%)	Deep (%)	Shallow (%)	Alpha helix (%)	Beta sheet (%)	Turn (%)	Random coil (%)	Mainly alpha (%)	Mainly beta (%)	Mixed alpha/beta (%)	L2S (%)	S2L (%)	S2S (%)
15–25	58	819	50.06	49.94	54.70	45.30	35.90	27.35	5.62	21.12	20.27	27.84	51.89	38.1	14.41	47.50
25–35	81	1143	59.14	40.86	57.66	42.34	30.45	39.28	6.39	15.31	15.84	25.11	56.78	36.83	15.05	48.12
35–45	86	1421	53.27	46.73	54.61	45.39	31.60	31.39	8.94	17.38	32.09	21.25	44.83	35.61	16.40	47.99
45–55	65	1078	53.62	46.38	55.29	44.71	28.01	34.14	9.18	15.40	28.39	21.11	49.75	35.06	16.33	48.61
55–65	64	995	57.79	42.21	52.76	47.24	27.14	34.47	8.74	18.09	28.64	21.11	49.75	29.55	19.70	50.75
65–75	47	931	51.34	48.66	50.27	49.73	29.75	29.22	9.67	17.62	35.45	26.10	35.66	36.31	15.04	48.66
75–85	42	918	53.38	46.62	56.10	43.90	38.24	30.17	5.34	17.21	32.24	20.48	46.73	33.66	16.23	50.11
85–95	56	1573	47.36	52.64	47.23	52.77	33.76	29.94	8.26	17.36	23.9	44.88	28.10	37.19	15.58	47.23
100	124	2492	50.76	49.24	61.16	38.84	32.95	30.82	10.23	15.25	26.12	33.15	38.56	38.32	14.69	46.99

Homology modelling was performed using MODELLER version 9.24 [74]. Potential templates were searched in the *pdb_95.bin* database (a cluster at 95% sequence identity in MODELLER) using *blastp* with the target sequence as input. Target-template alignments were manually inspected and a representative template selected per protein/per identity range based on the following criteria:

1. Target-template coverage >75%;
2. Template structure determined by crystallography with X-ray diffraction;
3. Best quality models were selected based on DOPE score.

Models generated were submitted to FoldX [55] for minimization and refinement. The structural similarity between homology models and protein experimental structures was then inspected using the root mean square deviation (RMSD) calculated by the *align* and *rms_cur* command in Pymol [75–77] and TM-score calculated by TM-align [78]. The full list of templates and targets used is available as Supplementary Materials.

### Generating high-quality protein models via AlphaFold2

The AlphaFold2 program developed by DeepMind dominated the 14th Critical Assessment of protein Structure Prediction (CASP14) [79], representing a significant advance in the field. The performance of predictive methods on the protein models generated via AlphaFold2 were also used as a benchmark in this study. The Uniprot sequences of the proteins in the S2648 dataset were used for construction of AlphaFold2 models. The installation of AlphaFold2 was introduced in their manuscript and is available in their *github* page. As suggested, the same database version *—max_template_date = 2020-05-14* was used in this work. Model quality was recorded in output files and determined by pLDDT values [80].

### Methods to predict effects of mutations on protein stability

Increased availability of high-quality mutation data and advances in computational approaches, particularly in machine learning, have supported and enabled the development of a range of computational tools aiming to understand how missense mutations affect protein folding and stability, which have been fundamental to unravelling molecular mechanisms of mutations leading to protein malfunction and diseases [81], also playing a role in cancer [82] and cancer risk [83], as well as drug resistance [17,18,84]. These developed methods can be divided into three main groups, without loss of generality: (i) tools based on energy function and dynamics simulations; (ii) knowledge-based and statistical; and (iii) machine learning methods. For this study, a representative set of ten structure-based methods was selected for assessment, including representatives of these three groups, for which either standalone packages or web-server interfaces were publicly available. These include:

#### Energy-based and dynamics

1. FoldX [55] is a well-established tool that uses empirical force fields and energy term calculations to estimate the effects of mutations on protein stability.
2. ENCoM [56] uses a coarse-grained normal mode analysis (NMA) approach to simulate effects of mutations on the conformational repertoire, and therefore dynamics, of protein structures.

#### Knowledge-based and statistical

1. SDM [42] adopts environment-specific substitution tables to generate a structure-based score for mutation propensity and effects, considering structurally aligned protein families.
2. DDGun [85] is a prediction method using a linear combination of sequence and structural fea-tures.

#### Machine learning

1. MAESTRO [46] uses statistical scoring functions and a multi-agent system to predict effects of mutations on protein stability.
2. I-Mutant 2.0 [51] is a Support Vector Machine (SVM)-based method to predict the change of protein stability upon mutation.
3. mCSM-Stability [44] uses the concept of graph-based signature to describe residue environments in protein structures and then train and test predictive models via supervised learning.
4. DUET [45] is an integrated computational approach to predict the ΔΔG values upon mutation that combines the prediction power of mCSM-Stability and SDM.
5. DynaMut1 [47] is a method that combines the graph-based signatures and NMA to give a consensus prediction of ΔΔG values upon mutation via supervised learning.
6. DynaMut2 [50] is an optimized version that also considers the global environment of the wild-type residues to estimate the conformational change upon residue substitution and train supervised learning methods.

We have also included the following currently available sequence-based methods as a baseline for comparison purposes and assess potential situations where performance deterioration by using homology models would suggest that sequence-based methods would be more adequate:

1. SAAFEC-SEQ [86] is a sequence-based method that applies a gradient boosting decision tree algorithm on protein sequence features descriptors, different physico-chemical factors and evolutionary knowledge to make predictions.
2. MUpro [87] is a method that considers a small window size on the neighbour of targeted residue to train a SVM-based predictive model.
3. I-Mutant 2.0 [51] is a sequence-based version of the I-Mutant package using SVM to predict mutation effects on protein stability.
4. DDGun-Seq [85] is a sequence-based version of DDGun using evolutionary information to predict ΔΔG values upon variants.

All the predictions were run at the same experimental pH and temperature as described in the S2648 dataset (available as Supplementary Materials), when these parameters were available. All the prediction tools were otherwise run with default settings. The *nr* database [88] was used for predictions of SAAFEC-SEQ. A detailed introduction of all methods used in this work is available in Table S1, including the implementation, relevant datasets and sources.

Mutations and homology models obtained in previous steps were systematically used and provided to the methods to predict effects of mutation on protein stability, with the experimental effect used as ground truth. Performance metrics were calculated including root mean square error (RMSE) and Pearson’s Correlation Coefficient (R), for regression purposes and Matthew’s Correlation Coefficient (MCC) and F1-score, for classification purposes. A description of metrics used can be found in Supplementary Materials.

### Characterizing method performance based on structural and sequence properties

To better characterize the performance of different methods, for different identity ranges, the mutation dataset was further divided for analysis purposes. This involved generating subsets of mutations based on structure- and sequence-based properties as follows:

#### Structure-based properties

Mutation subsets were divided for analysis purposes based on:

- Residue relative solvent accessibility (RSA—*buried versus exposed* residues), calculated using Biopython [89];
- Residue depth, calculated by the *msms* program in Biopython;
- Secondary structure type (SST), obtained from the DSSP algorithm [90,91]. Four main SST, namely alpha helix, beta sheet, turn and random coil, were considered in this work;
- CATH structural classification of proteins [92]. Three main types of structure classes, namely mainly alpha, mainly beta, and mixed alpha/beta, were considered in this work.

#### Sequence-based properties

Mutation groups were further obtained by grouping residues based on:

- Polarity, where wild-type residues are assigned to either polar (referred as P) including Q, N, S, T, R, H, K, D, E and C or hydrophobic/apolar (referred as H) including A, L, M, I, V, F, Y and W. Glycine residues were considered as a separate class. No mutations on Proline residues were identified in the S2648 dataset;
- Residue volume difference [93,94], classified based on the difference between wild-type and mutant residue volumes. Three groups were constructed, Large to Small (L2S), Same to Same (S2S) and Small to Large (S2L). Only when the difference was greater or equal to 30 Å^3^ would the mutation be inserted into the L2S or S2L groups [71];
- Stability of mutations was classified based on the experimentally measured effects. These were labelled as either stabilizing or destabilizing (based on the ΔΔG sign).

## Results

### Property distribution of the homology model datasets

**Figure 2:**
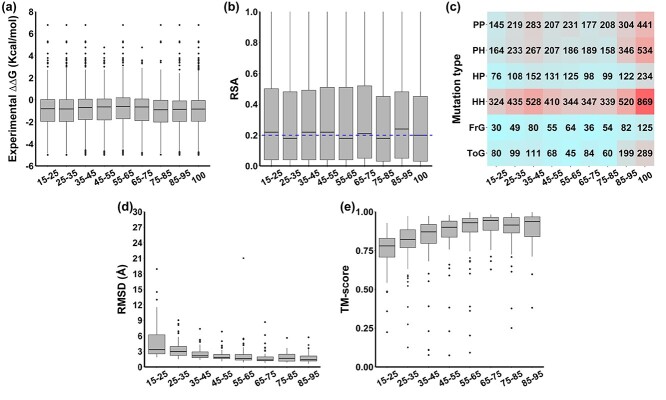
Distribution of (A) experimental ΔΔG values, (B) relative solvent accessibility (RSA), (C) mutation types, (D) root mean square deviation (RMSD) and (E) TM-score between homology models and experimental structures in eight homology model datasets of different identity levels. The RSA cutoff to define buried or exposed residues was 20% and shown as a blue dashed line in (B).

Eight homology model datasets at different identity levels, namely Iden15–25, Iden25–35, Iden35–45, Iden45–55, Iden55–65, Iden65–75, Iden75–85 and Iden85–95, were built and presented no significant differences in distributions of ΔΔG values (Figure 2A and Table S2, *P*-value = 0.38 via one-way ANOVA) when compared with the experimental dataset (Iden100), with significance decreasing with identity levels. The proportion of destabilizing (ΔΔG < 0) and stabilizing mutations (ΔΔG ≥ 0) in these datasets were consistent, with averages of 26% (sd = 0.03) and 74% (sd = 0.03), respectively, reflecting a natural bias towards detabilizing mutations in the S2648 dataset (Figure S1).

#### Distribution of structure-based properties

All datasets presented similar RSA distributions (Figure 2B). A general cutoff of 20% was used to define whether a residue was buried (53%) or exposed (47%) (Table 1), consistent with previous studies [95–97]. Residue depth distributions were also similar, ranging from 1.7 to 6.6 Å. In this study, we used 2.2 Å as a cutoff to distinguish deep (54%) and shallow (46%) residues, to achieve a relatively balanced split (Table 1). The distribution of mutations per SST is listed in Table 1. For all homology model datasets, two-thirds of mutations were located in alpha helix (32%) and beta sheet (32%) structures, with a smaller proportion of mutations in turns (8%), random coil (17%) or other secondary structures (11%). When looking at the distribution of protein structural classes, based on the CATH database, the majority of mutations were in proteins belonging to the alpha/beta class (45%), followed by mainly alpha (27%) and mainly beta (26%), with a small fraction of proteins (2%) labelled as ‘few secondary structures’ in CATH (Table 1).

#### Distribution of sequence-based properties

The distribution of mutation types showed that most mutations fall into the apolar-to-apolar (HH: 37%) category, followed by polar-to-polar (PP: 20%), and polar-to-apolar (PH: 20%) categories (Figure 2C). Eight percent of mutations were to Glycine and 27% of mutations to Alanine, as a reflection of experimental mutagenesis efforts. Most mutations involved wild-type and mutant residues of similar volumes (S2S: 49%), with smaller proportions involving the introduction of smaller (L2S: 35%) or larger residues (S2L: 16%) (Table 1).

#### Distribution of RMSD and TM-score

We observed that higher sequence identities led in general to lower RMSDs and higher TM-score (Table 2, Figure 2D and E), consistent with previous analyses [61]. When sequence identity reaches around 40%, almost two-thirds of the models in the dataset obtained RMSD of around 1 Å and TM-score of above 0.75. Only one model in the dataset Iden55–65 had a high RMSD value of around 21 Å. However, this has been kept to mimic the real-world scenario of application of homology models.

**Table 2:** Template description for homology model datasets.

Identity level (%)	Mean RMSD (Å)	Mean TM-score	Mean actual identity (%)	Mean coverage (%)	Mean template resolution (Å)
15–25	4.90	0.75	21.19	86.49	1.91
25–35	3.68	0.79	30.73	88.13	1.93
35–45	2.50	0.82	39.86	91.01	1.85
45–55	2.19	0.86	49.11	92.76	1.87
55–65	2.29	0.86	59.95	94.51	1.78
65–75	1.89	0.90	69.43	96.48	1.74
75–85	1.86	0.87	79.74	95.96	1.92
85–95	1.74	0.89	89.82	95.11	1.78

#### Distribution of target-template identity, coverage and quality of models

Target-template identity distributions for each dataset are depicted in Supplementary Figure S2A, with average values consistently in the middle of the range. Most models presented target-template coverage higher than 85% (Supplementary Figure S2B), suggesting that the target-template coverage was less limiting than target-template identity when electing a template for homology modelling. The quality of all homology models shared similar distribution in each identity range (Supplementary Figure S2C).

### Predictive performance trends on homology model datasets

#### Overall performance

**Figure 3:**
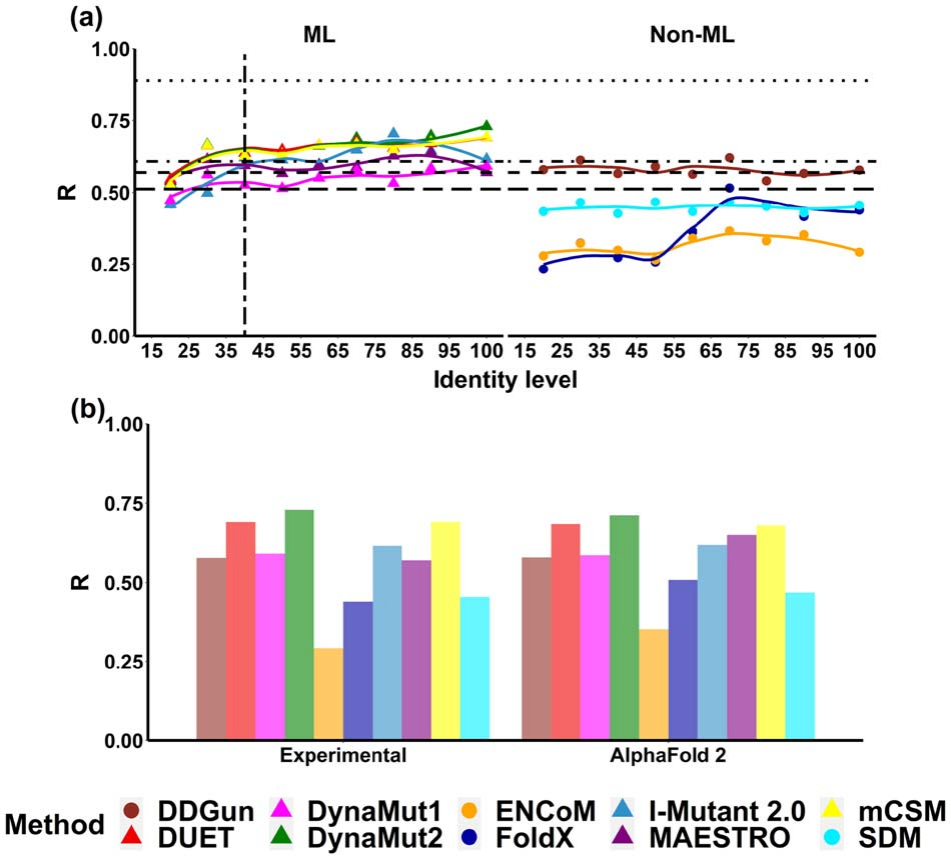
Overall performance trends based on Pearson’s correlation coefficient (*R*) of ten methods predicting mutation effects on protein stability, namely DDGun (brown), DUET (red), DynaMut1 (pink), DynaMut2 (green), ENCoM (orange), FoldX (blue), I-Mutant 2.0 (light blue), MAESTRO (purple), mCSM-Stability (yellow) and SDM (cyan). The *R* values and their trends on homology models are represented in dots and lines, respectively. A vertical long-dashed line indicates the proposed identity cutoff for homology modelling, whereas the horizontal lines are the baseline performance of four sequence-based methods, namely SAAFEC-SEQ (dotted), MUpro (dot-dashed), I-Mutant (dashed) and DDGun (long-dashed).

We observed that, in general, predictive performance of the evaluated methods increases with target-template identity, which was consistent for both regression and classification tasks (Figure 3 and Supplementary Figure S3). Alternatively, we observed a consistent performance deterioration on the task of predicting mutation effects on stability for all structure-based methods, particularly in machine learning based methods and FoldX, when the sequence identity of the homology modelling template dropped. Interestingly, the predictive performance of most methods studied on AlphaFold2 models is close to those obtained on experimental structures (Figure 3B).

Based on the performance trend shown in Figure 3A, a proposed sequence identity cutoff for DynaMut2, DUET and mCSM-Stability was around 40%. The regression performances of these three prediction methods presented a sharp decreasing trend when the sequence identity was less than 40% (with *R* values dropping from 0.63 to 0.53 and RMSE increasing from 1.22 to 1.34 Kcal/mol), while keeping relatively stable performance for identity levels higher than 40%. Interestingly, below 40% identity the performance of structure-based methods deteriorated as low as that of sequence-based methods, indicating that the latter would be recommended in the absence of higher identity templates for homology modelling. The same result can be observed for classification tasks (MCC = 0.21–0.36, F1-score = 0.32–0.45). The SAAFEC-SEQ, as a sequence-based benchmark, showed the highest correlation among all methods (*R* = 0.89).

DynaMut1, Maestro and I-Mutant presented a similar behaviour when sequence identity reaches the 40% mark, with *R* values decreasing from 0.62 to 0.46 and RMSE increasing from 1.21 to 1.44 Kcal/mol (Figure 3 and Supplementary Figure S3). The performance of these three methods also deteriorated below the baseline from sequence-based methods when identity is under this threshold. FoldX had the largest degree of variation on performance when sequence identity changes and did not show robust performance for models built with templates with sequence identity below 70%. As for the performance trends of DDGun, SDM and ENCoM, there was no clear sequence identity cutoff for these three methods, with performance varying substantially. Only DDGun exceeded the baseline performance of sequence-based methods. To remove the bias caused by outliers and homologs in the original S2648 dataset, a detailed performance report is showcased in Supplementary Table S3. Similar identity cutoffs for machine learning methods were determined and no significant difference between the results before and after removing homolog structures was observed.

### Performance trends based on structural properties

We further assessed how the performance of different predictive methods vary based on the structural properties of proteins and residues involved. In this study, we considered buried versus exposed residues, residues in different secondary structures and in proteins of different structural classes derived from CATH.

#### Exposed versus buried residues

**Figure 4:**
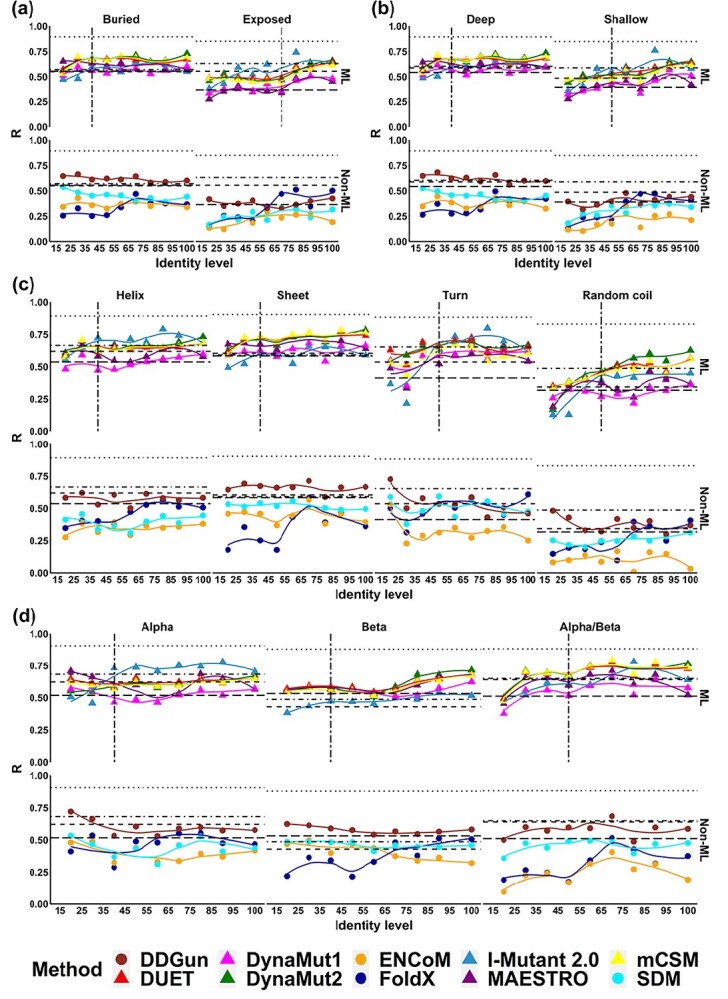
Performance trends based on Pearson’s correlation coefficient (*R*) of ten methods with mutations grouped based on four structure-based properties, namely (A) relative solvent accessibility (RSA), (B) residue depth, (C) secondary structure types and (D) structural class based on CATH. The performance trends of two main types of methods, namely machine learning based (ML) and Statistical/Energy function based (Non-ML), were displayed respectively. The RSA cutoff of 20% was used to determine buried or exposed residues. The residue depth cutoff of 2.2 Å was used to determine deep or shallow residues. Four secondary structure types, namely alpha helix, beta sheet, turn and random coil, were considered in this study. Three structural classifications, namely mainly alpha, mainly beta and mixed alpha/beta, were analysed.

The assessed methods tended to perform better on buried than exposed residues. When sequence identity was lower than 40%, I-Mutant, DUET, mCSM-Stability and DynaMut2 presented a larger drop in performance on buried residues (R dropped from 0.67 to 0.55) (Figure 4A) than on exposed residues (R dropped from 0.48 to 0.45), even though overall performance on the former mutation group was still higher. Classification performance on buried residues for these methods showed a sharp reduction when sequence identity was less than 50% (MCC dropped from 0.41 to 0.20) (Supplementary Figure S4). Among these four methods, I-Mutant showed the best classification performance on exposed mutations (MCC up to 0.55), whereas most could not reach the baseline performance of sequence-based methods. This trend was also observed for the remaining methods. When it comes to the statistical/energy function methods, only FoldX showed large performance deterioration when sequence identity dropped.

#### *Shallow* versus *deep residues*

For globular proteins, residue depth can distinguish between buried residues just under the surface and those near the protein core region [98–100]. As expected, prediction performance trends on deep and shallow residues correlated with buried and exposed residues (Figure 4A and B). DynaMut2, mCSM-Stability, DUET and I-Mutant shared a similar trend, with performance deteriorating below identity cutoff of 40% and 50% for deep and shallow residue mutations, respectively (consistent with F1-score for classification tasks—Supplementary Figure S5). FoldX shows the larger performance variation below 70% identity for both deep and shallow mutations (Figure 4B). Little performance variation until 40% identity was observed for DynaMut1, Maestro, SDM, ENCoM and DDGun.

#### Secondary structure

In general, methods performed better on structured regions (alpha helices, beta sheets and turns) than on unstructured ones (random coil). DynaMut2, mCSM-Stability and DUET shared similar performance trends, performing well up to 40% identity for mutations on alpha helix and beta sheet and 50% for turn and random coil (Figure 4C and S6), outperforming sequence-based methods. Similar performance trends were observed in DynaMut1, Maestro, as well as I-Mutant, revealing lower sequence identity demands on alpha helix and beta sheet, which were higher for FoldX. I-Mutant had the highest performance deterioration in turn conformations (R dropped from 0.62 to 0.36, Figure 4C). Similar identity cutoff on alpha helices and beta sheets can be observed in classification tasks, with MCC ranging from 0.27 to 0.47 (Figure S6). SDM, ENCoM and DDGun showed no distinguishable trend based on secondary structure.

#### CATH classification

When assessing performance based on protein structural classification, mainly alpha and mixed alpha/beta proteins presented clearer trends (Figure 4D), with a drop in performance below 40% identity. DynaMut2, mCSM-Stability and DUET showed a steadier performance on mainly alpha proteins, with larger drops for mainly beta and mixed alpha/beta. These were consistent for machine learning based methods and FoldX, with I-Mutant showing the most significant performance deterioration on classification tasks (Figure S7) and no discernible trend for ENCoM and DDGun. There was no obvious identity cutoff for DynaMut1 and Maestro in the mainly alpha group (Figure 4D). Performance deterioration was more pronounced in mainly beta and mixed alpha/beta proteins.

### Performance trends based on sequence-based properties

For sequence-based properties, this study focused on mutations on different amino acid residue types, residue volume differences, and the direction of stability change upon mutation.

**Figure 5:**
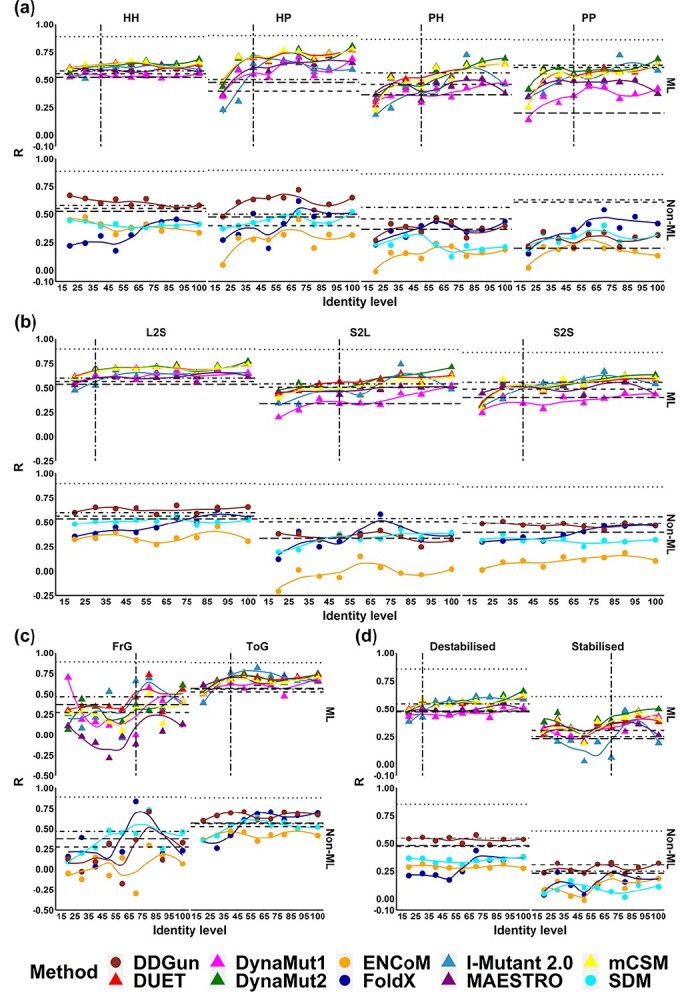
Performance trends based on Pearson’s correlation coefficient (*R*) of ten methods with mutations grouped based on four sequence-based properties, namely (A) mutation type based on the change of polarity, (B) change of residue volume, (C) mutations related to Glycine and (D) mutation effects on protein stability.

#### Mutation types

When assessing performance trends based on mutation types (to and from polar and apolar residues), inter-group mutations (HP and PH) presented, in general, a more pronounced performance deterioration below 40–50%, particularly for the PH type for all methods (Figure 5A and S8). Only a small performance deterioration was observed for HH mutations for most methods, apart from FoldX. Better performance and reliability on inter-group mutations could be influenced by the natural distribution of mutation effects around mildly destabilizing mutations, which were enriched in S2648, and would influence particularly machine learning methods. Consistent with that, no trends for SDM, ENCoM and DDGun were observed.

#### Change of residue volume

Figure 5B and S9 depict the performance trends when categorizing mutations based on volume changes between wild-type and mutant residues. In general, performance deteriorated less for this category, with most methods being outperformed by sequence-based alternatives for L2S volume mutations below 40%. Methods seemed more robust for large to small changes (L2S), with DynaMut2, mCSM-Stability and DUET, maintaining performance down to 30% identity. On the other hand, performance deteriorated more quickly for S2L mutations (around 50% identity). No clear trends were identified for SDM, ENCoM as well as DDGun.

#### Mutations to/from Glycine

Performance trends were consistent for mutations to Glycine (ToG), with most methods only showing a sharp decrease in correlation around 40% identity (Figure 5C and S10), while outperforming sequence-based methods even beyond this threshold. Alternatively, for mutations from Glycine (FrG), a substantial variation in performance was observed, with a peak in performance around 70%, which might be due to the limited number of mutations in this category (5% mutations), in comparison with mutations to Glycine, which were often used in mutagenesis experiments (8% mutations).

#### Effect of mutation on thermodynamics stability of proteins

In general, all methods performed better on destabilizing mutations, which is consistent with previous observations [71,101,102]. For destabilizing mutations (Figure 5D and S11), most machine learning methods only presented performance deterioration below 30% identity. These methods have been shown before to perform better on this mutation type, a bias that was introduced due to the natural distribution of mutation effects, which has been attempted to be corrected with the use of hypothetical reverse mutations [47,48,54,86]. No trends were observed for SDM, ENCoM or DDGun. Consistently, a sharper decrease in performance was observed for stabilizing mutations below 70% identity.

## Discussion and conclusion

To better understand how *in silico* mutation analysis tools behave in the absence of high-resolution experimental protein structures, we used homology models at different identity levels to systematically test the performance of ten widely used computational tools to predict mutation effects on protein stability. We found that when target-template identity for homology modelling drops below 40%, there is an evident performance deterioration for structure-based methods, especially for the machine learning based approaches, a point where sequence-based methods might be preferable. It has been previously reported that in order to build reliable models of a protein of interest, the structure used as a template should share at least 30% sequence identity to the target [60]. The identity cutoff identified in this work is more conservative than this accepted rule-of-thumb and can be further validated in the future as more thermodynamics data becomes available. Although some small differences between the prediction using homology models and one using experimental structure were noticed, we think this mainly results from the prediction variance of the prediction methods themselves, which was reported in a test on structural sensitivity [103]. We also found that the predictive performance on AlphaFold2 models was highly consistent with that on experimental structures for most tools. It represents an important breakthrough in the field of protein structure prediction as the community actively seeks the explore its limitations and adapt it for other applications.

When assessing different mutation categories based on structural and sequence-based properties, we found that the identity cutoffs varied from the overall threshold described above. The reason for this may be a native bias of the prediction methods, with predictors performing better on residues that are not solvent exposed, deeper in the structures and for destabilizing mutations, consistent with previous observations [71]. Alternatively, structure-based methods performed worse on exposed residues, random coil conformations, less frequent mutations (e.g. from Glycine) and stabilizing mutations, requiring a higher identity cutoff (50–70%) when using homology models.

In brief, this work showed that, as sequence identity of the template decreased, the performance of the tools deteriorated beyond the performance of sequence-based tools. As expected, this was more pronounced for exposed residues and mutations in random coils, where the largest deviations in structure modelling are likely to be found. We found that a minimum target-template identity cutoff around 40% was necessary for robust performance of structure-based tools when using homology models as inputs, larger than the minimum 30% sequence identity threshold often used as a rule-of-thumb for homology modelling. We expect that these insights will help guide the accurate use and interpretation of these computational tools in the absence of experimental structures moving forward.

**Key Points/Highlights**

- We present the first systematic study assessing how methods to predict stability changes upon mutations cope in the absence of high-resolution experimental protein structures.
- This work provides a detailed guideline for *in silico* mutation analysis, which will assist users in appropriately using and interpreting prediction results, which could assist in the study of mutations in protein design and in genetic diseases.
- This work first applied protein structural models from traditional homology modelling and AlphaFold2 modelling to mutation effect analysis.

## Supplementary Material

Supplementary_materials_bbac025

Supplementary_Target-template_list_bbac025

Supplementyary_S2648_dataset_bbac025

## Data Availability

Datasets used in the analyses described have been made available as Supplementary Materials.

## Notes

**Qisheng Pan** is a PhD candidate at the School of Chemistry and Molecular Bioscience of the University of Queensland. His research interests include characterising and predicting mutation effects on protein function and structure.

**Thanh Binh Nguyen** is a postdoctoral research fellow at the Baker Institute of Heart and Diabetes and the University of Queensland. Her interest is in computational biology and understanding protein structures, function and their interactions.

**David B. Ascher** is Deputy Director of Biotechnology at The University of Queensland, and head of Computational Biology and Clinical Informatics at the Baker Institute and Systems and computational Biology at Bio21 Institute. He is interested is in developing and applying computational tools to assist leveraging clinical and omics data for drug discovery and personalised medicine.

**Douglas E.V. Pires** is a Senior Lecturer in Digital Health with the School of Computing and Information Systems at the University of Melbourne and group leader at Bio21 Institute. He is a computer scientist and bioinformatician specialising in machine learning and AI and the development of tools to analyse omics data.
