# Supplementary material for: Systematic evaluation of computational tools to predict the effects of mutations on protein stability in the absence of experimental structures
Source: Brief Bioinform. 2022 Feb 21;23(2):bbac025. doi: 10.1093/bib/bbac025 (PMC9155634; doi:10.1093/bib/bbac025)
Supplement: Supplementary_materials_bbac025 [file supplementary_materials_bbac025.pdf]

SUPPLEMENTARY MATERIALS

Systematic evaluation of computational tools to predict the effects of mutations on protein stability in the absence of experimental structures

Qisheng Pan[1,2,3], Thanh Binh Nguyen[1,2,3], David B. Ascher[1,2,3,4,*], Douglas E.V. Pires[1,2,3,5,*]

[1]Computational Biology and Clinical Informatics, Baker Heart and Diabetes Institute, Melbourne VIC 3004, Australia
[2]Department of Biochemistry and Molecular Biology, University of Melbourne, Melbourne, VIC 3010, Australia
[3]Systems and Computational Biology, Bio21 Institute, University of Melbourne, 30 Flemington Rd, Parkville VIC 3052, Australia
[4]Department of Biochemistry, University of Cambridge, 80 Tennis Ct Rd, Cambridge CB2 1GA, UK
[5]School of Computing and Information Systems, University of Melbourne, Melbourne, VIC 3053, Australia

*To whom correspondence should be addressed D.E.V.P. Email: douglas.pires@unimelb.edu.au. Correspondence may also be addressed to D.B.A. Tel: +61 90354794; Email: david.ascher@unimelb.edu.au.

Performance metrics calculation

Four metrics to evaluate regression and classification performance respectively were used in this work, using the Python sci-kit learn and NumPy packages.

Here are the formulas for these four metrics.

1. Root mean square error (RMSE)

$$RMSE = \sqrt{\frac{(Y_{pred} - Y_{true})^2}{N}}$$

2. Pearson's Correlation Coefficient (R)

$$R = \frac{\sum(Y_{pred} - \overline{Y}_{pred})(Y_{true} - \overline{Y}_{true})}{\sqrt{\sum(Y_{pred} - \overline{Y}_{pred})^2}\sqrt{\sum(Y_{true} - \overline{Y}_{true})^2}}$$

Consider the following example confusion matrix.

		Predicted condition	
		Positive	Negative
Actual condition	Positive	True Positive (TP)	False Negative (FN)
	Negative	False Positive (FP)	True Negative (TN)

3. Matthew Correlation Coefficient (MCC)

$$MCC = \frac{TP \times TN - FP \times FN}{\sqrt{(TP + FP)(TP + FN)(TN + FP)(TN + FN)}}$$

4. F1-score

$$F1 = \frac{2}{recall^{-1} + precision^{-1}} = \frac{TP}{TP + \frac{1}{2}(FP + FN)}$$

TABLES

Table S1. Description of the methods to predict effects of mutation on protein stability.

Method	Type	Dataset	Implementation	Source
mCSM-Stability	Machine learning	S2648, S1925, *p53*	Structure-based method.<br>It can be run on the web server.<br>It usually takes less than 1 minute for each mutation, depending on the protein size.	http://biosig.unimelb.edu.au/mcsm/stability
DUET	Machine learning	S2648, *p53*	Structure-based method.<br>It can be run on the web server.<br>It usually takes less than 1 minute for each mutation, depending on the protein size.	http://structure.bioc.cam.ac.uk/duet
MAESTRO	Machine learning	S2648, S1925, S1765,	Structure-based method.<br>It can be run either on the web server or via a standalone software.<br>The standalone software usually takes less than 30 seconds for each mutation, depending on the protein size.	http://biwww.che.sbg.ac.at/MAESTRO
DynaMut	Machine learning	S2648, S350	Structure-based method.<br>It can be run on the web server, and provides 5 prediction results including mCSM-stability, DUET, ENCoM, SDM, and DynaMut.<br>It usually takes less than 5 minutes for each mutation, depending on the protein size.	http://biosig.unimelb.edu.au/dynamut/
DynaMut2	Machine learning	S2648, S276, S173	Structure-based method.<br>It can be easily accessed on the web server or via an API.<br>It usually takes less than 1 minute for each mutation, depending on the protein size.	http://biosig.unimelb.edu.au/dynamut2/
I-Mutant 2.0	Machine learning	2087	Structure- and sequence-based method.	https://folding.biofold.org/cgi-bin/i-

		mutations	It can be run either on the web server or via Python scripting.<br>The standalone package usually takes less than 30 seconds for each mutation, depending on the protein size.	mutant2.0.cgi
SAAFEC-SEQ	Machine learning	S2648, S276, *p53*, PTEN, TPMT	Sequence-based method.<br>It can be run either on the web server or via Python scripting.<br>The standalone package usually takes around 20 minutes for each mutation if the *nr* database is used, depending on the protein size.	http://compbio.clemson.edu/SAAFEC-SEQ/index.php
MUpro	Machine learning	S1615, S388	Sequence-based methods.<br>It can be run on the web server.<br>It usually takes less than 1 minute for each mutation, depending on the protein size.	http://mupro.proteomics.ics.uci.edu/
SDM	Statistical	S2648, S350, *p53*, S140	Structure-based method.<br>It can be run on the web server.<br>It usually takes less than 1 minute for each mutation, depending on the protein size.	http://marid.bioc.cam.ac.uk/sdm2
DDGun	Statistical	S2648, VariBench, Broom, *p53*	Structure- and sequence-based method.<br>It can be run via Python scripting.<br>The standalone package usually takes less than 3 minutes for each mutation, depending on the protein size.	http://folding.biofold.org/ddgun/predictions.tar.gz
FoldX	Energy function	/	Structure-based method.<br>It provides a standalone package for mutation effect prediction.<br>It usually takes less than 1 minute for each mutation, depending on the protein size.	http://foldxsuite.crg.eu/
ENCoM	Energy function	/	Structure-based method.<br>It can be run on the web server.<br>It usually takes less than 1 minute for each mutation, depending on the protein size.	http://bcb.med.usherbrooke.ca/encom

Table S2. The statistics of experimental ΔΔG values (Kcal/mol) in each Iden dataset

Identity (%)	Mean ΔΔG	Median ΔΔG	Minimum ΔΔG	Maximum ΔΔG	Standard deviation
15–25	-0.91	-0.81	-5.00	6.80	1.53
25–35	-0.94	-0.83	-5.00	6.80	1.59
35–45	-0.85	-0.70	-5.00	6.80	1.52
45–55	-0.84	-0.65	-5.00	4.75	1.52
55–65	-0.72	-0.60	-4.99	6.80	1.55
65–75	-0.87	-0.65	-5.00	4.75	1.52
75–85	-1.02	-0.90	-4.98	6.80	1.49
85–95	-1.06	-0.86	-5.00	6.80	1.47
100	-1.02	-0.84	-5.00	6.80	1.49

Table S3. Performance comparison among raw, removing outliers, and removing homologs of homology model datasets in each identity range.

Methods	Identity range	RMSE (Kcal/mol)			Pearson's Correlation Coefficient			MCC			F1-score		
		raw	Remove homologs	Remove 10% outliers	raw	Remove homologs	Remove 10% outliers	raw	Remove homologs	Remove 10% outliers	raw	Remove homologs	Remove 10% outliers
DynaMut2	15-25	1.340	1.374	0.994	0.530	0.541	0.676	0.220	0.238	0.273	0.317	0.335	0.342
DynaMut2	25-35	1.233	1.269	0.925	0.666	0.652	0.772	0.319	0.306	0.374	0.399	0.396	0.438
DynaMut2	35-45	1.228	1.241	0.888	0.637	0.627	0.772	0.361	0.375	0.421	0.454	0.456	0.501
DynaMut2	45-55	1.248	1.247	0.915	0.632	0.635	0.774	0.333	0.339	0.404	0.433	0.442	0.484
DynaMut2	55-65	1.230	1.229	0.901	0.662	0.677	0.776	0.408	0.412	0.473	**0.505**	0.500	0.558
DynaMut2	65-75	1.159	1.154	0.880	0.689	0.709	0.802	0.354	0.376	0.410	0.457	0.476	0.499
DynaMut2	75-85	1.162	1.194	0.891	0.650	0.668	0.759	**0.416**	0.432	0.471	0.469	0.489	0.515
DynaMut2	85-95	**1.075**	1.066	0.783	**0.697**	0.687	0.812	0.379	0.349	0.415	0.444	0.421	0.479
DynaMut2	100	1.027	1.025	0.766	0.729	0.734	0.816	0.405	0.415	0.431	0.517	0.529	0.537
FoldX	15-25	2.197	2.254	1.452	0.232	0.206	0.506	0.192	0.195	0.261	0.448	0.467	0.491
FoldX	25-35	2.198	2.153	1.305	0.323	0.312	0.612	0.285	0.307	0.342	0.500	0.528	0.543
FoldX	35-45	2.589	2.442	1.301	0.273	0.325	0.595	0.274	0.314	0.334	0.491	0.532	0.534
FoldX	45-55	2.809	3.008	1.297	0.257	0.213	0.602	0.270	0.267	0.328	0.492	0.501	0.535
FoldX	55-65	2.287	2.016	1.216	0.363	0.396	0.655	0.357	0.360	0.403	**0.574**	0.577	0.607
FoldX	65-75	**1.820**	1.744	1.072	**0.516**	0.518	0.736	0.374	0.385	0.433	0.567	0.597	0.612
FoldX	75-85	2.046	1.968	1.132	0.454	0.488	0.696	**0.388**	0.429	0.428	0.545	0.577	0.577
FoldX	85-95	2.125	2.176	1.042	0.416	0.405	0.727	0.344	0.342	0.379	0.498	0.497	0.529

FoldX	100	2.076	1.819	1.056	0.438	0.513	0.736	0.379	0.395	0.435	0.532	0.551	0.575
I-Mutant 2.0	15-25	1.440	1.434	1.063	0.458	0.497	0.649	0.280	0.346	0.368	0.429	0.479	0.492
I-Mutant 2.0	25-35	1.438	1.408	1.053	0.498	0.530	0.680	0.283	0.337	0.370	0.433	0.481	0.497
I-Mutant 2.0	35-45	1.219	1.197	0.817	0.620	0.629	0.794	0.464	0.499	0.571	0.583	0.615	0.666
I-Mutant 2.0	45-55	1.245	1.235	0.844	0.614	0.627	0.786	0.456	0.472	0.543	0.586	0.590	0.651
I-Mutant 2.0	55-65	1.268	1.237	0.853	0.597	0.629	0.781	0.420	0.480	0.513	0.579	0.624	0.645
I-Mutant 2.0	65-75	1.175	1.179	0.805	0.648	0.649	0.802	0.489	0.542	0.553	0.614	0.652	0.664
I-Mutant 2.0	75-85	**1.092**	1.192	0.727	**0.704**	0.671	0.857	**0.531**	0.556	0.628	**0.636**	0.657	0.709
I-Mutant 2.0	85-95	1.178	1.157	0.814	0.645	0.651	0.807	0.417	0.415	0.481	0.545	0.547	0.595
I-Mutant 2.0	100	1.219	1.259	0.832	0.616	0.601	0.789	0.408	0.417	0.490	0.526	0.541	0.589
MAESTRO	15-25	1.324	1.342	0.996	0.539	0.545	0.677	0.262	0.260	0.311	0.453	0.460	0.481
MAESTRO	25-35	1.270	1.228	0.932	0.615	0.637	0.745	0.342	0.343	0.395	0.504	0.513	0.538
MAESTRO	35-45	1.234	1.187	0.877	0.594	0.613	0.739	0.375	0.380	0.421	0.537	0.549	0.568
MAESTRO	45-55	1.280	1.202	0.925	0.567	0.606	0.722	0.351	0.372	0.407	0.531	0.548	0.569
MAESTRO	55-65	1.258	1.254	0.899	0.595	0.605	0.742	0.385	0.408	0.439	**0.568**	0.587	0.602
MAESTRO	65-75	1.251	1.167	0.898	0.588	0.638	0.742	0.349	0.378	0.401	0.521	0.550	0.555
MAESTRO	75-85	1.178	1.155	0.855	0.628	0.663	0.763	0.390	0.404	0.426	0.522	0.528	0.547
MAESTRO	85-95	**1.168**	1.116	0.838	**0.638**	0.660	0.761	**0.410**	0.408	0.452	0.543	0.542	0.576
MAESTRO	100	1.268	1.197	0.893	0.570	0.625	0.744	0.334	0.364	0.385	0.487	0.512	0.524
DynaMut1	15-25	1.441	1.459	1.104	0.472	0.469	0.610	0.182	0.187	0.223	0.413	0.432	0.439
DynaMut1	25-35	1.393	1.360	1.048	0.562	0.559	0.688	0.282	0.294	0.323	0.490	0.509	0.518

DynaMut1	35-45	1.365	1.339	0.985	0.526	0.507	0.679	0.307	0.306	0.356	0.514	0.522	0.549
DynaMut1	45-55	1.381	1.315	1.009	0.514	0.526	0.668	0.241	0.245	0.289	0.473	0.486	0.507
DynaMut1	55-65	1.350	1.343	0.976	0.551	0.560	0.697	0.316	0.313	0.360	**0.547**	0.547	0.577
DynaMut1	65-75	**1.310**	1.220	0.962	0.577	0.606	0.722	0.313	0.353	0.377	0.520	0.555	0.565
DynaMut1	75-85	1.377	1.381	1.017	0.532	0.546	0.680	**0.331**	0.338	0.374	0.509	0.519	0.545
DynaMut1	85-95	1.324	1.304	0.990	**0.584**	0.583	0.690	0.238	0.227	0.262	0.426	0.418	0.450
DynaMut1	100	1.363	1.322	0.994	0.592	0.608	0.737	0.331	0.361	0.381	0.507	0.536	0.552
ENCoM	15-25	1.642	1.659	1.280	0.279	0.254	0.334	0.070	0.073	0.087	0.327	0.345	0.345
ENCoM	25-35	1.689	1.648	1.291	0.323	0.290	0.432	0.192	0.213	0.195	0.413	0.436	0.425
ENCoM	35-45	1.612	1.574	1.198	0.299	0.251	0.403	0.184	0.156	0.202	0.427	0.420	0.445
ENCoM	45-55	1.628	1.574	1.206	0.265	0.232	0.391	0.124	0.097	0.142	0.390	0.382	0.411
ENCoM	55-65	**1.581**	1.591	1.171	0.340	0.324	0.414	0.179	0.167	0.178	**0.450**	0.442	0.460
ENCoM	65-75	1.587	1.513	1.195	**0.365**	0.351	0.423	0.190	0.235	0.206	0.421	0.457	0.443
ENCoM	75-85	1.654	1.674	1.277	0.331	0.338	0.436	**0.201**	0.178	0.223	0.416	0.402	0.441
ENCoM	85-95	1.642	1.616	1.227	0.353	0.337	0.405	0.155	0.105	0.157	0.366	0.327	0.377
ENCoM	100	1.682	1.663	1.278	0.292	0.287	0.363	0.137	0.164	0.140	0.371	0.393	0.385
DUET	15-25	1.301	1.320	0.968	0.545	0.556	0.692	0.299	0.320	0.358	0.457	0.471	0.494
DUET	25-35	1.198	1.201	0.883	0.662	0.658	0.783	0.397	0.388	0.445	0.540	0.543	0.572
DUET	35-45	1.176	1.174	0.844	0.638	0.629	0.775	0.387	0.398	0.430	0.530	0.543	0.564
DUET	45-55	1.170	1.152	0.854	0.648	0.651	0.781	0.399	0.381	0.442	0.553	0.545	0.584
DUET	55-65	1.161	1.160	0.844	0.665	0.674	0.779	0.441	0.449	0.484	**0.594**	0.600	0.621

DUET	65-75	1.129	1.097	0.842	**0.676**	0.697	0.795	0.414	0.431	0.442	0.565	0.587	0.584
DUET	75-85	1.135	1.148	0.838	0.654	0.671	0.783	**0.460**	0.463	0.503	0.567	0.569	0.602
DUET	85-95	**1.106**	1.097	0.802	0.670	0.661	0.796	0.408	0.397	0.436	0.529	0.520	0.553
DUET	100	1.087	1.106	0.799	0.691	0.683	0.802	0.444	0.430	0.465	0.567	0.565	0.584
mCSM	15-25	1.303	1.333	0.964	0.525	0.537	0.663	0.232	0.271	0.277	0.309	0.329	0.329
mCSM	25-35	1.206	1.216	0.896	0.663	0.657	0.746	0.296	0.274	0.318	0.360	0.350	0.376
mCSM	35-45	1.188	1.188	0.855	0.627	0.621	0.744	0.338	0.320	0.393	0.417	0.394	0.464
mCSM	45-55	1.186	1.169	0.874	0.632	0.637	0.744	0.340	0.335	0.393	0.422	0.415	0.466
mCSM	55-65	1.176	1.176	0.860	0.664	0.679	0.751	0.392	0.373	0.427	**0.482**	0.455	0.511
mCSM	65-75	1.141	1.107	0.866	0.668	0.700	0.769	0.335	0.345	0.363	0.425	0.439	0.442
mCSM	75-85	1.136	1.142	0.849	0.649	0.673	0.748	**0.425**	0.410	0.452	0.441	0.437	0.466
mCSM	85-95	**1.089**	1.073	0.800	**0.674**	0.667	0.768	0.363	0.314	0.383	0.411	0.368	0.433
mCSM	100	1.084	1.098	0.798	0.692	0.688	0.773	0.398	0.372	0.419	0.447	0.434	0.468
SDM	15-25	1.561	1.561	1.188	0.433	0.447	0.619	0.192	0.220	0.250	0.429	0.451	0.468
SDM	25-35	1.584	1.546	1.198	0.465	0.485	0.645	0.267	0.289	0.334	0.480	0.506	0.526
SDM	35-45	1.559	1.540	1.201	0.426	0.432	0.599	0.226	0.283	0.288	0.458	0.514	0.501
SDM	45-55	**1.501**	1.452	1.145	**0.467**	0.494	0.640	0.305	0.336	0.376	0.520	0.553	0.571
SDM	55-65	1.557	1.576	1.153	0.433	0.439	0.626	0.303	0.345	0.387	**0.533**	0.564	0.585
SDM	65-75	1.516	1.498	1.175	0.466	0.483	0.629	**0.312**	0.340	0.374	0.525	0.557	0.569
SDM	75-85	1.541	1.533	1.178	0.452	0.476	0.634	0.297	0.343	0.369	0.485	0.521	0.538
SDM	85-95	1.565	1.555	1.194	0.429	0.432	0.599	0.243	0.272	0.302	0.439	0.461	0.486

SDM	100	1.545	1.553	1.175	0.455	0.455	0.633	0.248	0.263	0.307	0.450	0.472	0.496
DDGun	15-25	1.352	1.359	0.991	0.579	0.580	0.745	0.330	0.344	0.382	0.531	0.553	0.568
DDGun	25-35	1.359	1.342	0.998	0.613	0.605	0.767	**0.334**	0.347	0.387	0.530	0.555	0.568
DDGun	35-45	1.362	1.342	0.978	0.566	0.545	0.742	0.284	0.284	0.344	0.502	0.519	0.544
DDGun	45-55	1.339	1.278	0.982	0.590	0.597	0.751	0.311	0.318	0.364	0.525	0.542	0.563
DDGun	55-65	1.402	1.376	1.015	0.563	0.572	0.737	0.300	0.321	0.361	**0.536**	0.553	0.579
DDGun	65-75	**1.269**	1.229	0.933	**0.621**	0.621	0.772	0.305	0.309	0.356	0.520	0.540	0.555
DDGun	75-85	1.390	1.389	1.009	0.540	0.551	0.719	0.290	0.312	0.347	0.484	0.503	0.527
DDGun	85-95	1.371	1.372	0.997	0.566	0.538	0.733	0.234	0.224	0.286	0.431	0.425	0.474
DDGun	100	1.352	1.359	0.987	0.578	0.570	0.741	0.263	0.267	0.313	0.459	0.473	0.499

* The values with red and bold label represent the best regression/classification performance of a certain predictive method across all identity ranges.

* Identity 100 represents the experimental inputs.

FIGURES

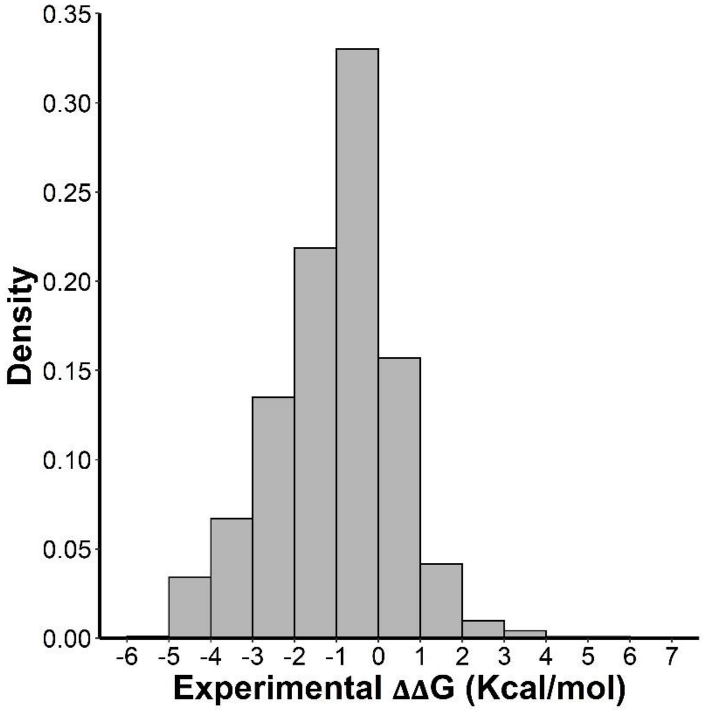

Figure S1. Distribution of the change of Gibbs free energy (ΔΔG) for the S2648 dataset.

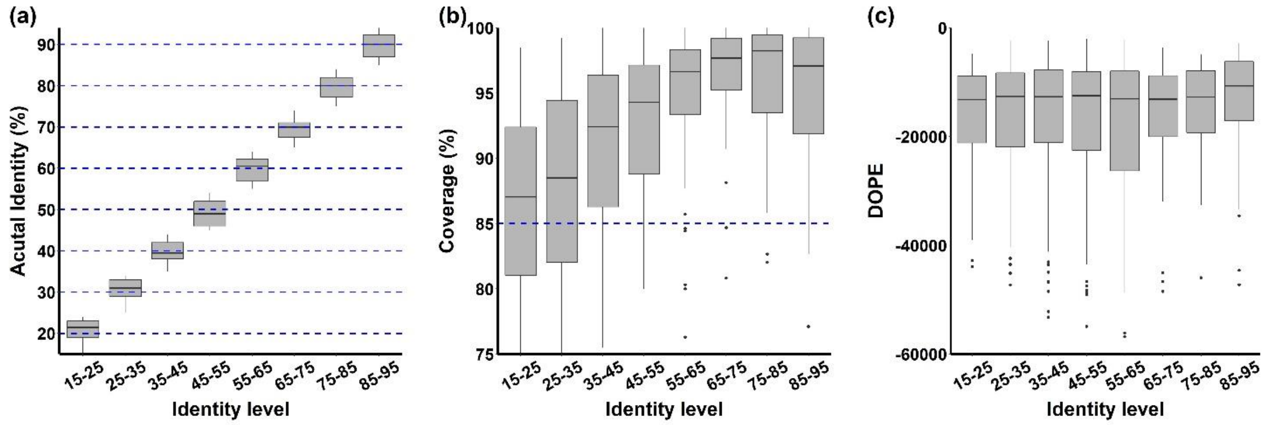

Figure S2. Distribution of (a) actual identity in each identity range, (b) coverage of homology models and (c) model quality assessed via DOPE score. Blue dashed lines in (a) showed the designed identity for each dataset, while in (b) presented the 85% coverage cutoff for all the models.

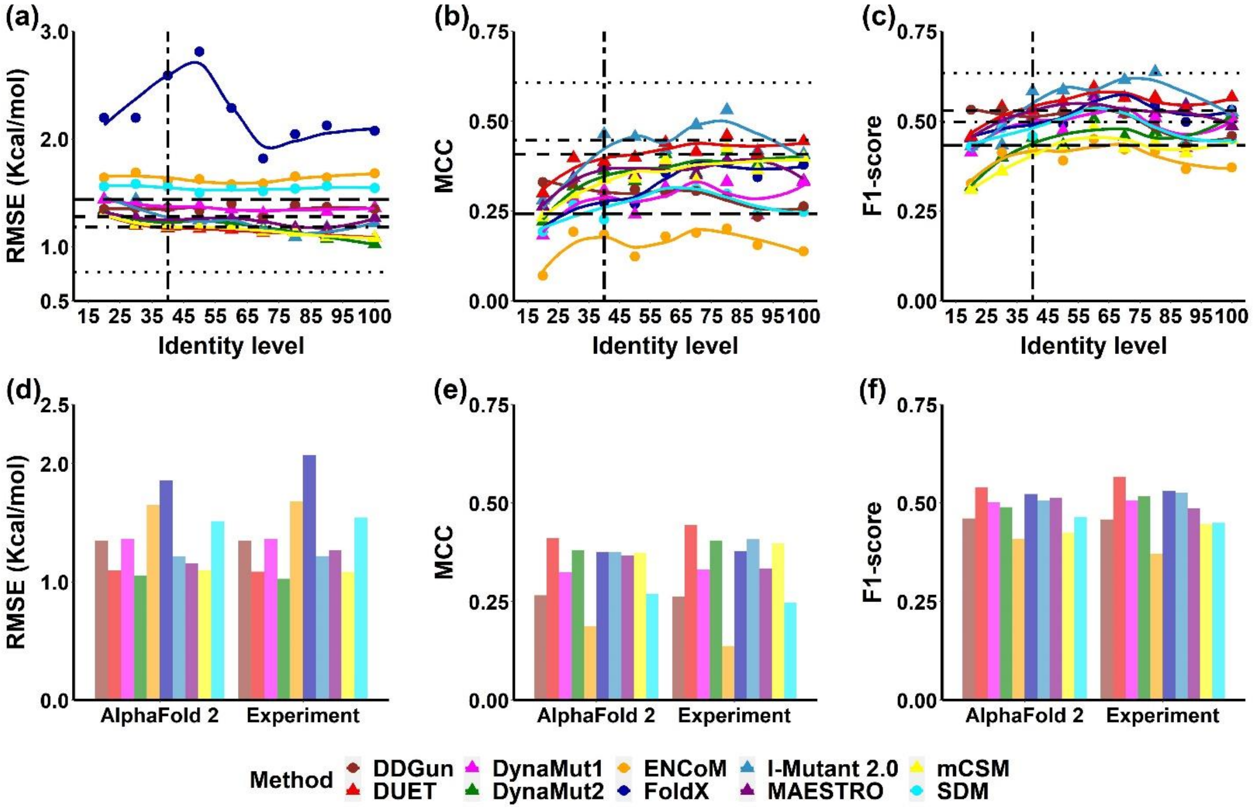

Figure S3. Overall performance trends based on RMSE, MCC and F1-score of ten methods predicting mutation effects on protein stability, namely DDGun (brown), DUET (red), DynaMut1 (pink), DynaMut2 (green), ENCoM (orange), FoldX (blue), I-Mutant 2.0 (light blue), MAESTRO (purple), mCSM-Stability (yellow), and SDM (cyan) on homology models (a-c) and AlphaFold2 models (d-f). The metrics values and their trends are represented in dots and lines, respectively. A vertical long-dashed line indicates the proposed identity cutoff for homology modelling, while the horizontal lines are the baseline performance of four sequence-based methods, namely SAAFEC-SEQ (dotted), MUpro (dot-dashed), I-Mutant (dashed), and DDGun (long-dashed).

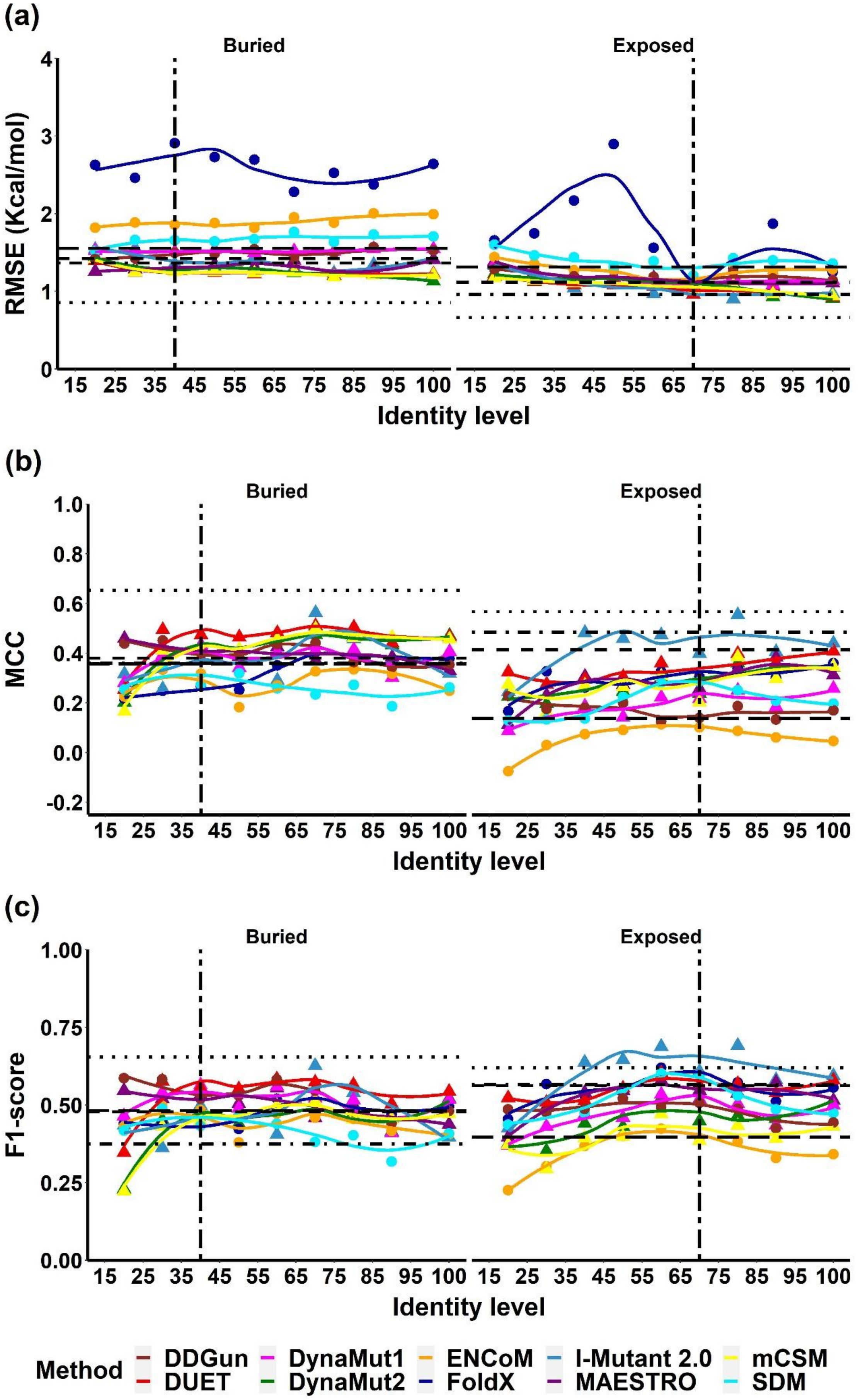

Figure S4. Performance trends based on (a) RMSE, (b) MCC and (c) F1-score for ten methods used to predict mutation effects on protein stability using homology models as input structures on buried *vs.* exposed residues.

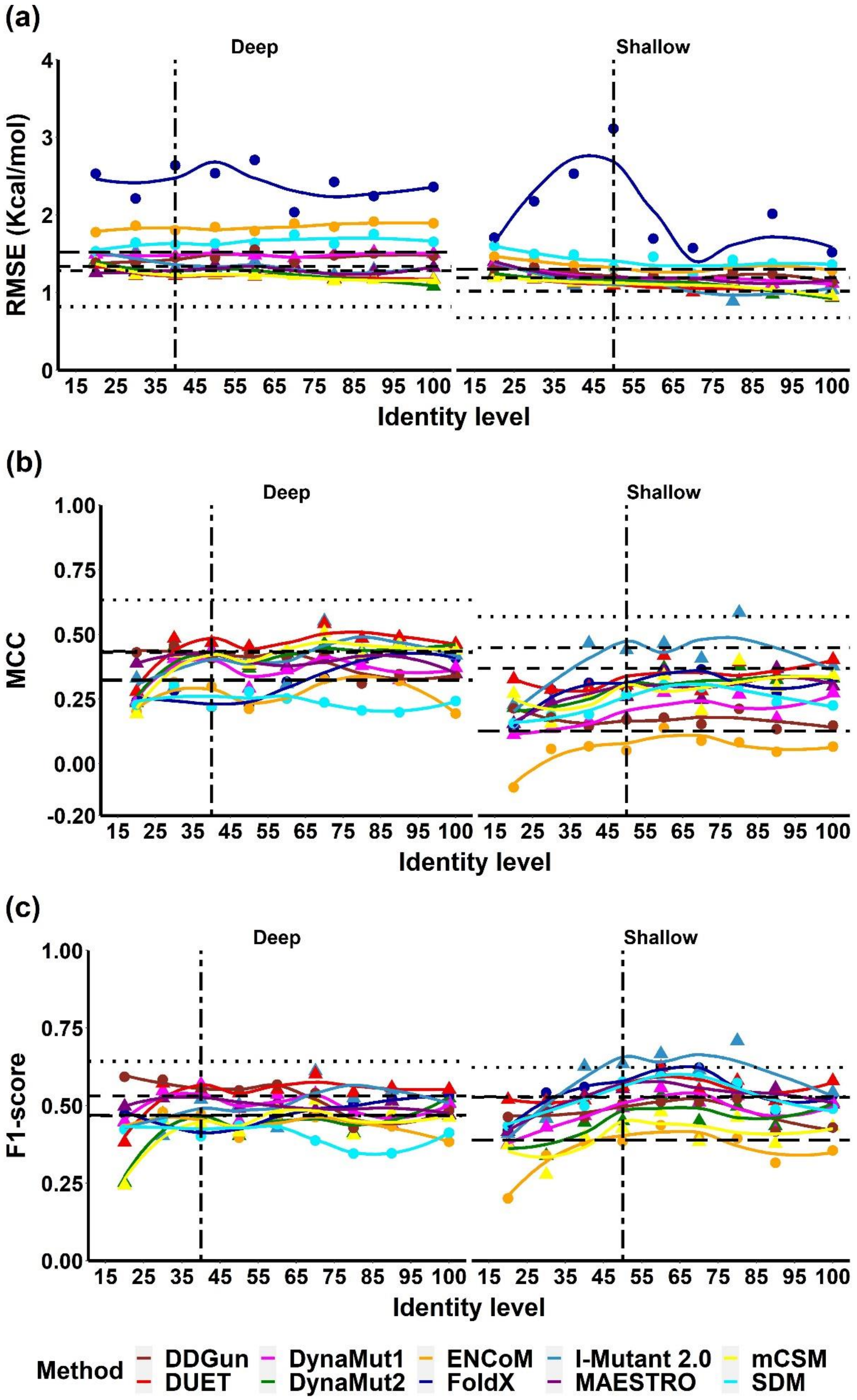

Figure S5. Performance trends based on (a) RMSE, (b) MCC and (c) F1-score for ten methods used to predict mutation effects on protein stability using homology models as input structures on deep *vs.* shallow residues.

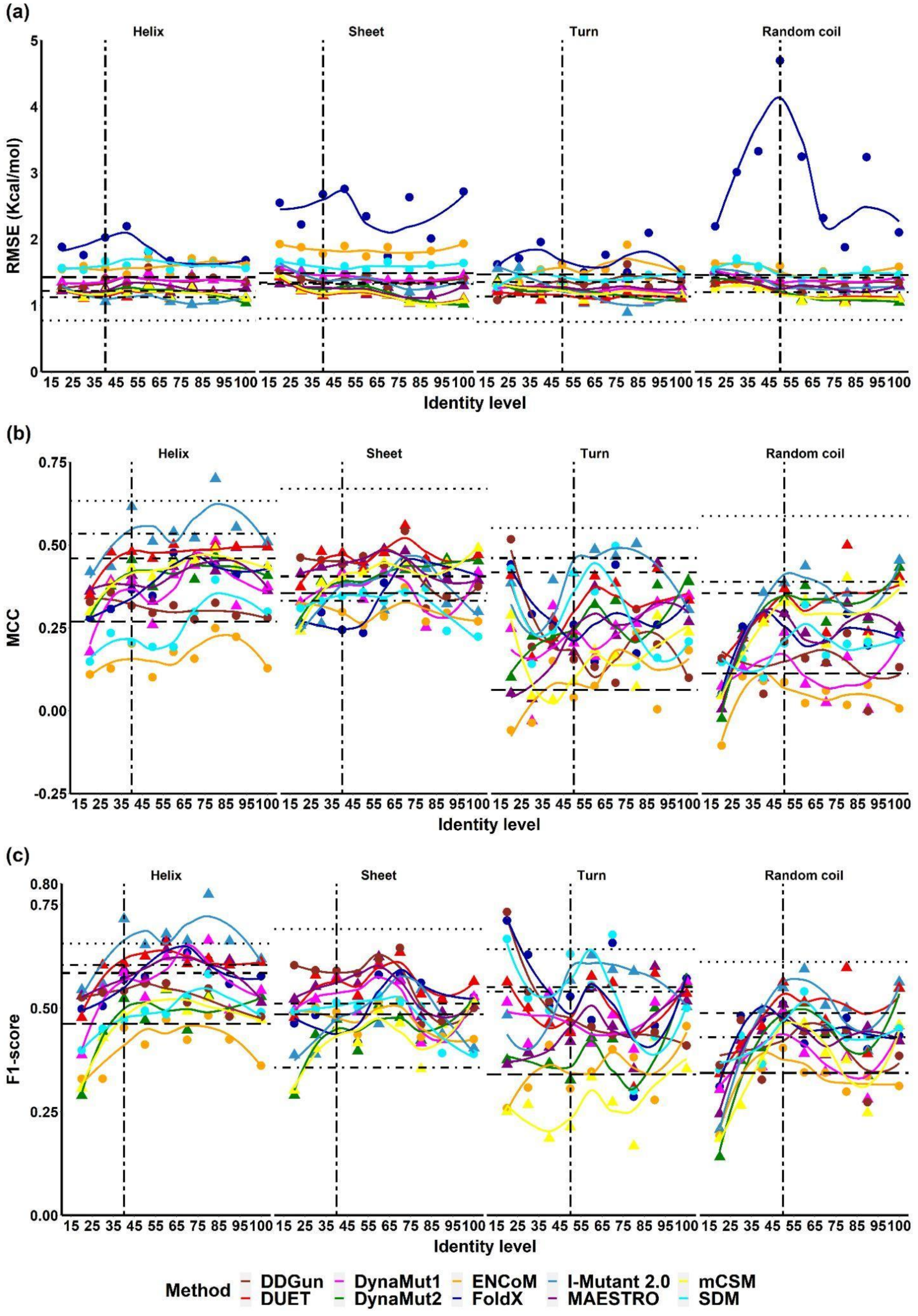

Figure S6. Performance trends based on (a) RMSE, (b) MCC and (c) F1-score for ten methods used to predict mutation effects on protein stability using homology models as input structures on residues in four secondary structure types.

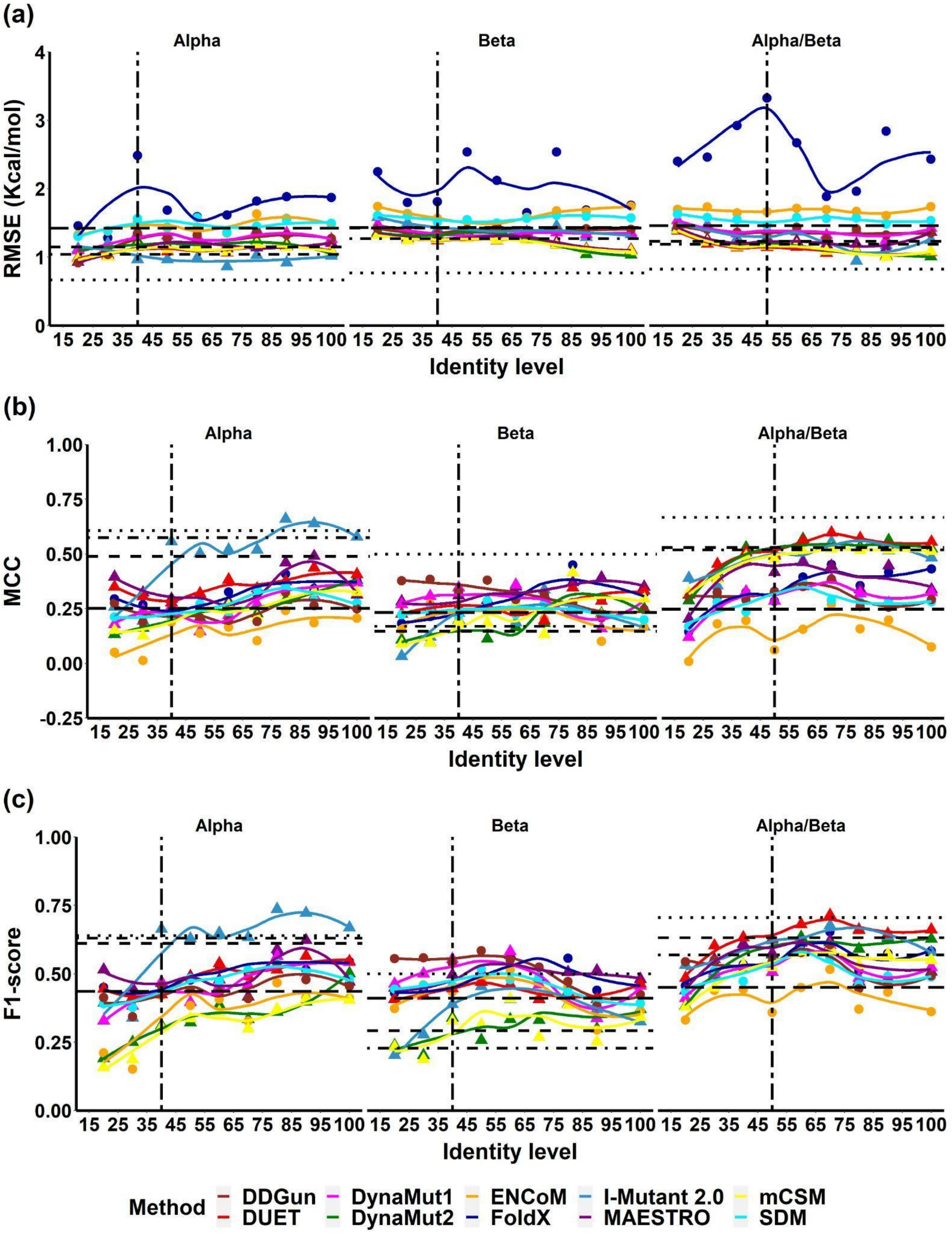

Figure S7. Performance of (a) RMSE, (b) MCC, and (c) F1-score of ten methods to predict mutation effects on protein stability with homology models as input structures on different secondary structure composition classified by the CATH.

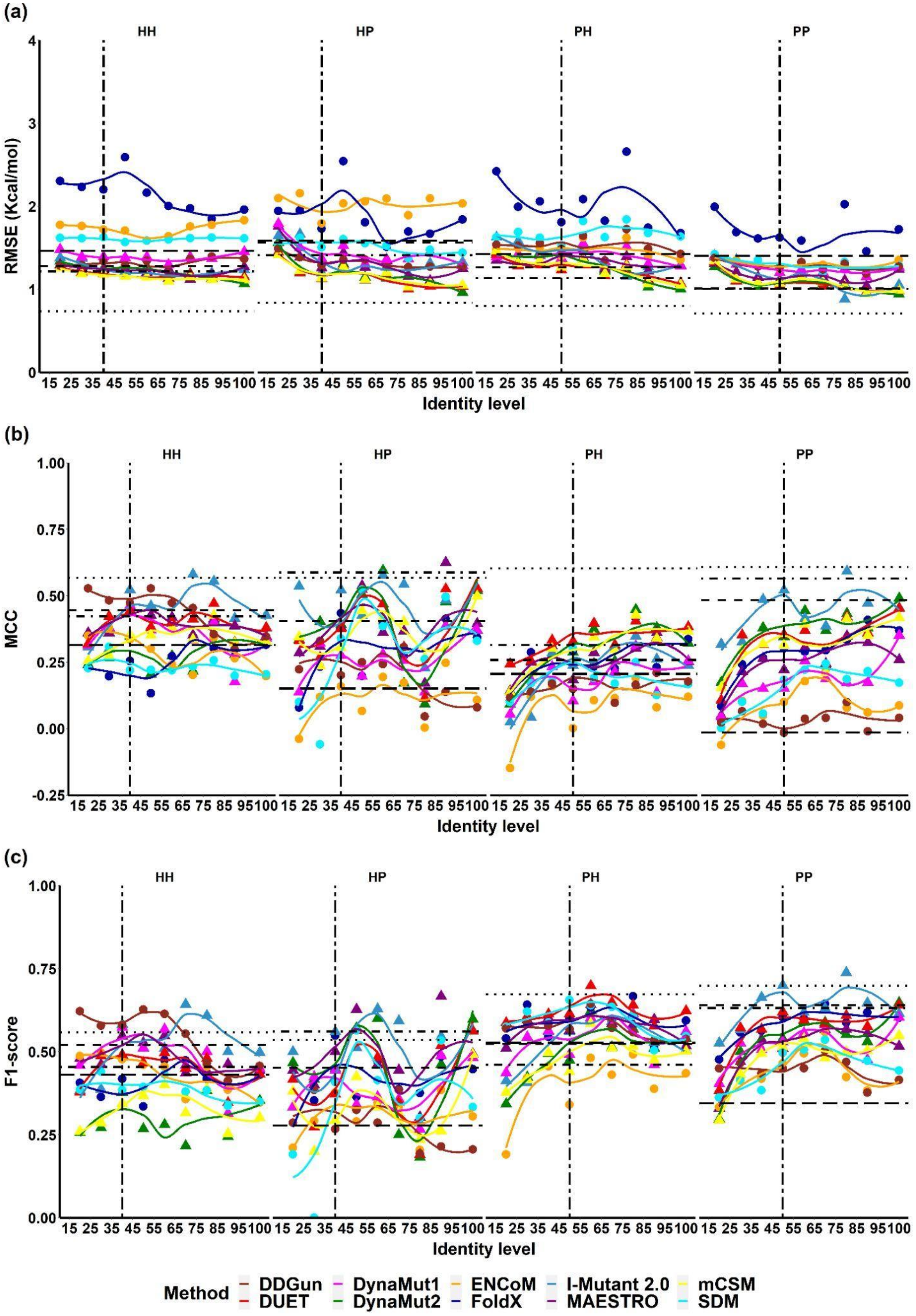

Figure S8. Performance trends based on (a) RMSE, (b) MCC and (c) F1-score for ten methods used to predict mutation effects on protein stability using homology models as input structures on different mutation types based on change of polarity.

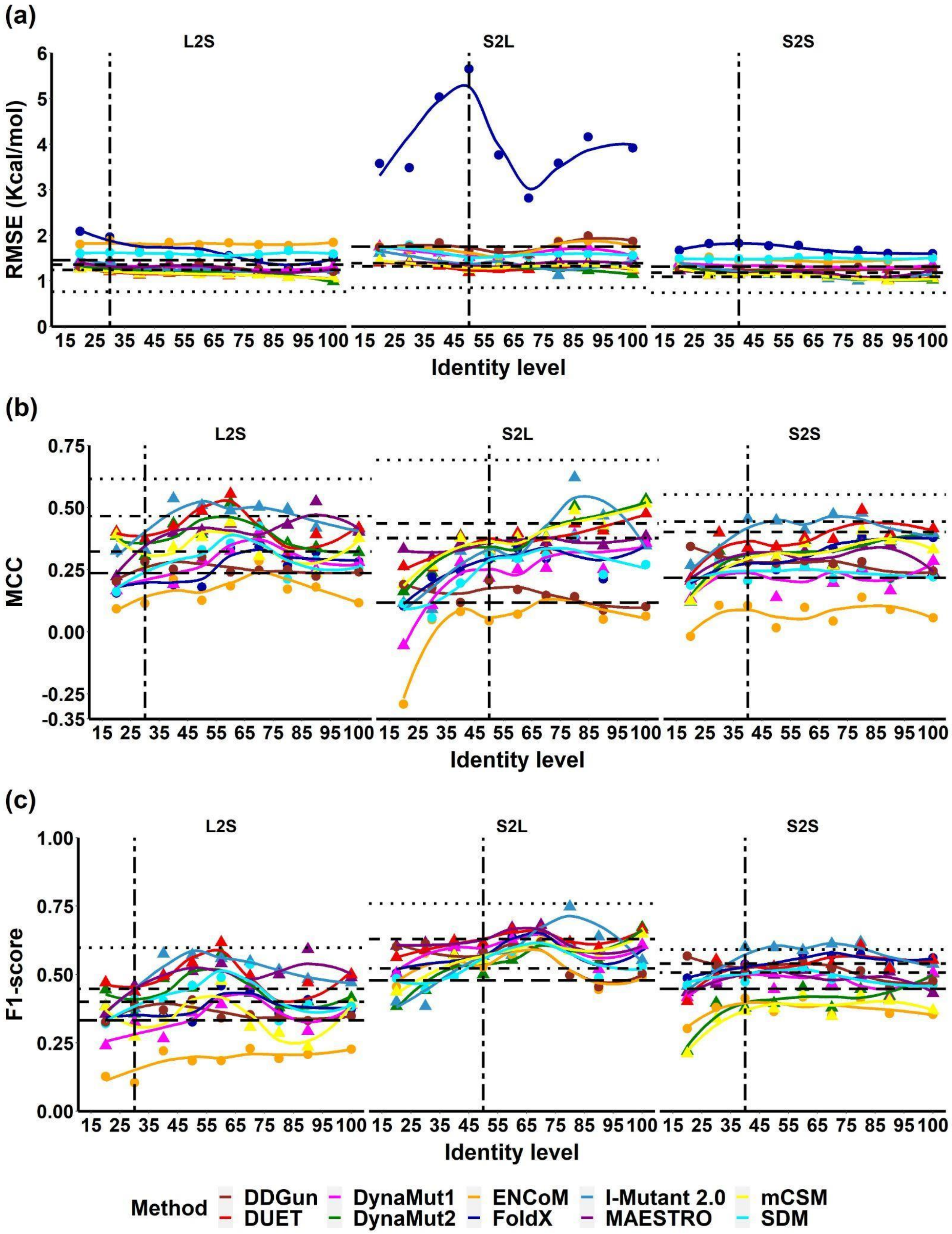

Figure S9. Performance trends based on (a) RMSE, (b) MCC and (c) F1-score for ten methods used to predict mutation effects on protein stability using homology models as input structure on different groups of change of residue volume.

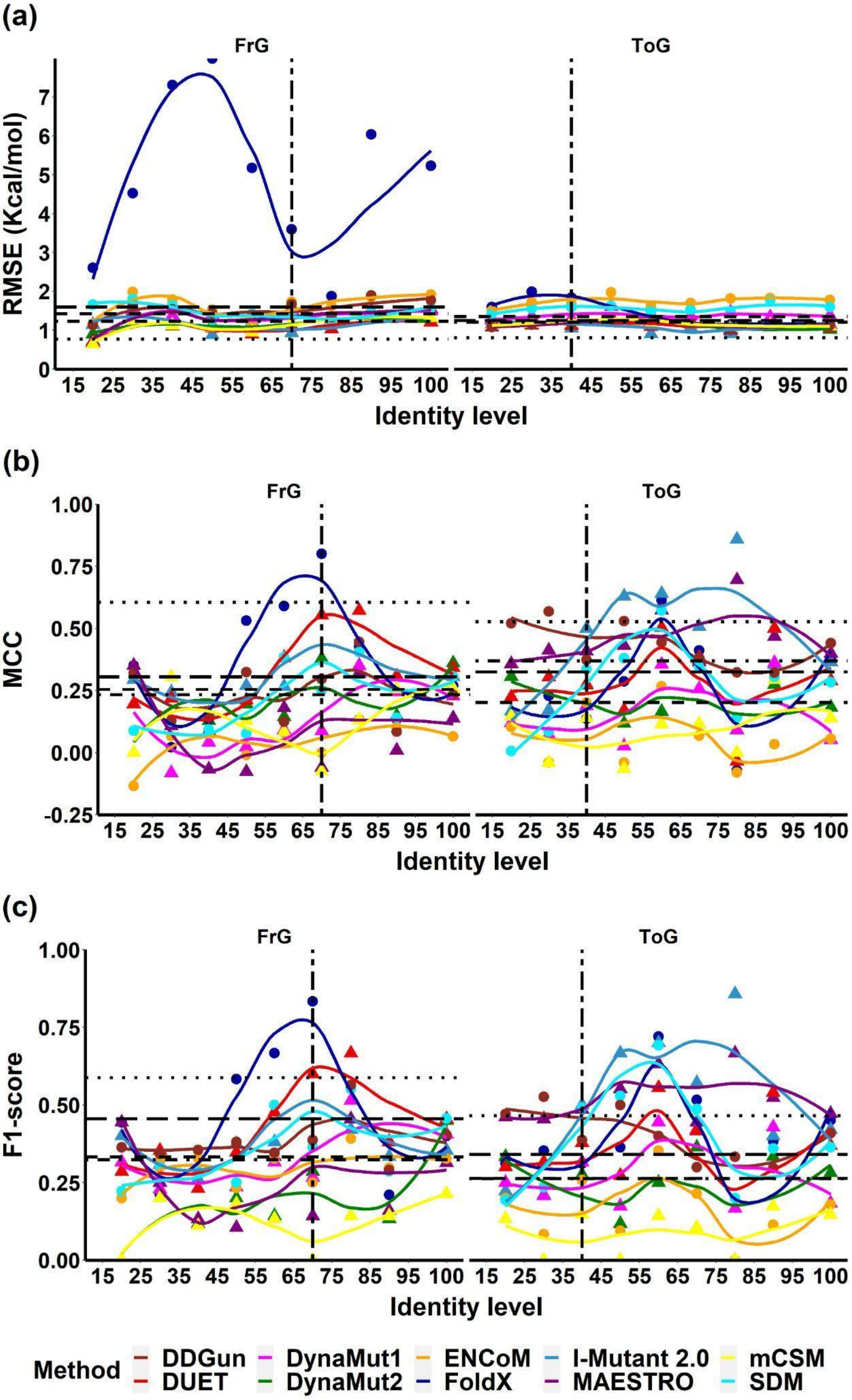

Figure S10. Performance trends based on (a) RMSE, (b) MCC and (c) F1-score for ten methods used to predict mutation effects on protein stability using homology models as input structure on different mutations related to Glycine.

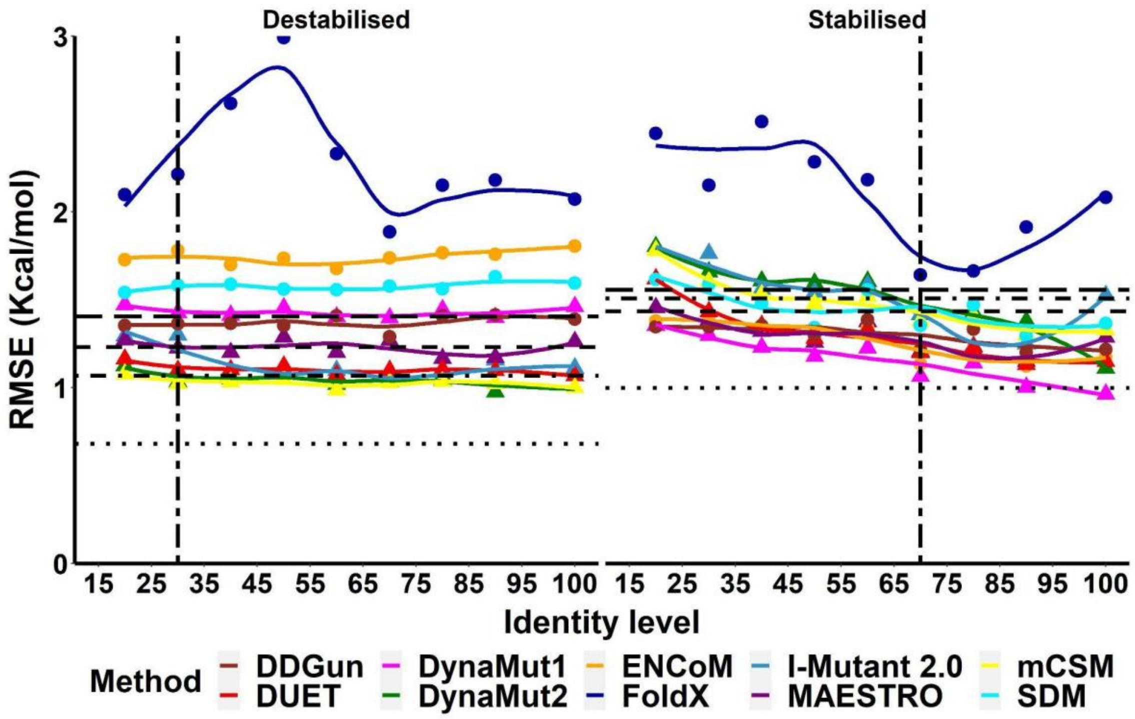

Figure S11. Performance trends based on RMSE for ten methods used to predict mutation effects on protein stability using homology models as input structure on different mutation effects on protein stability.
